# Perinatal OCD - A Lived Experience

**DOI:** 10.1192/j.eurpsy.2022.98

**Published:** 2022-09-01

**Authors:** D. Wilson

**Affiliations:** Maternal OCD, Support, London, United Kingdom

## Abstract

• Diana discusses the terror of perinatal ocd undiagnosed with four small children ages six years and under

• Diana explains fearing seeking help and seeing a psychiatrist knowing she would have to disclose her thoughts and images she encountered daily

• She pinpoints what moved her forward and stresses the importance of revisiting ocd when it is not in your life

• She talks of hope and through successful CBT and Citalipram, has been free of the disorder for twenty years

**Disclosure:**

No significant relationships.

